# Impact of glycemic control on the association of endothelial dysfunction and coronary artery disease in patients with type 2 diabetes mellitus

**DOI:** 10.1186/s12933-021-01257-y

**Published:** 2021-03-13

**Authors:** Shuai Chen, Ying Shen, Yong-Hua Liu, Yang Dai, Zhi-Ming Wu, Xiao-Qun Wang, Chen-Die Yang, Le-Ying Li, Jing-Meng Liu, Li-Ping Zhang, Wei-Feng Shen, Ri Ji, Lin Lu, Feng-Hua Ding

**Affiliations:** 1 Department of Cardiology, Rui Jin Hospital, Shanghai Jiao Tong University School of Medicine, 197 Rui Jin Road II, Shanghai, 200025 People’s Republic of China; 2 Department of Cardiology, Bao Shan People’s Hospital, Baoshan, Yunnan Province China; 3 Department of Ultrasound, Rui Jin Hospital, Shanghai Jiao Tong University School of Medicine, Shanghai, China

**Keywords:** Flow-mediated dilation, Type 2 diabetes mellitus, Coronary artery disease, Atherosclerosis

## Abstract

**Background:**

We investigated whether glycemic control affects the relation between endothelial dysfunction and coronary artery disease in patients with type 2 diabetes mellitus (T2DM).

**Methods:**

In 102 type 2 diabetic patients with stable angina, endothelial function was evaluated using brachial artery flow-mediated dilation (FMD) with high-resolution ultrasound, and significant stenosis of major epicardial coronary arteries (≥ 50% diameter narrowing) and degree of coronary atherosclerosis (Gensini score and SYNTAX score) were determined. The status of glycemic control was assessed by blood concentration of glycated hemoglobin (HbA1c).

**Results:**

The prevalence of significant coronary artery stenosis (67.9% vs. 37.0%, P = 0.002) and degree of coronary atherosclerosis (Gensini score: 48.99 ± 48.88 vs. 15.07 ± 21.03, P < 0.001; SYNTAX score: 15.88 ± 16.36 vs. 7.28 ± 10.54, P = 0.003) were higher and FMD was lower (6.03 ± 2.08% vs. 6.94 ± 2.20%, P = 0.036) in diabetic patients with poor glycemic control (HbA1c ≥ 7.0%; n = 56) compared to those with good glycemic control (HbA1c < 7.0%; n = 46). Multivariate regression analysis revealed that tertile of FMD was an independent determinant of presence of significant coronary artery stenosis (OR = 0.227 95% CI 0.056–0.915, P = 0.037), Gensini score (β =  − 0.470, P < 0.001) and SYNTAX score (β =  − 0.349, P = 0.004) in diabetic patients with poor glycemic control but not for those with good glycemic control (P > 0.05).

**Conclusion:**

Poor glycemic control negatively influences the association of endothelial dysfunction and coronary artery disease in T2DM patients.

## Background

Endothelial dysfunction occurs early in the chain of atherosclerotic process and is mainly caused by systemic effects of common risk factors for coronary artery disease [1, 2]. In current clinical practice, endothelial function is often evaluated non-invasively using flow-mediated dilation (FMD) (i.e., endothelial-dependent vasodilation) of the brachial artery with high-resolution ultrasonography [3, 4], which represents predominantly physiologic or pathophysiologic response to nitric oxide-mediated endothelial activation [5, 6]. Previous studies have frequently shown an inverse correlation between impaired brachial artery FMD and severity of coronary artery disease [7–10]. Netomo et al. [11] found that impaired endothelial function induced rapid progression of the culprit lesion, resulting in severe coronary artery stenosis as well as plaque vulnerability. More importantly, assessment of FMD provides useful information on risk stratification of patients with coronary artery disease and prediction of cardiovascular events [12–14].

Type 2 diabetes mellitus (T2DM) is independently associated with an increased risk for cardiovascular diseases that is primarily due to the early development of advanced atherosclerotic vascular changes [15]. Recently, endothelial dysfunction has received increasing attention as a potential contributor to the pathogenesis of cardiovascular diseases in type 2 diabetic patients [12, 13, 16]. The mechanisms underlying this phenomenon remain unclear but are likely to be multifactorial. The Hoorn study suggested that T2DM and endothelial dysfunction may interact with regard to the pathogenesis of cardiovascular events in a bidirectional association [13]. T2DM leads to endothelial dysfunction via, amongst others, dyslipidemia, formation of advanced glycated end products, intra-endothelial accumulation of glucose, increased oxidative stress, and low-grade inflammatory responses [17]. On the other hand, endothelial dysfunction causes or aggravates T2DM by impairing the timely access of glucose or insulin to target tissues [18].

It is well-recognized that chronic hyperglycemia could further accelerate the development of endothelial dysfunction and the pathological process of atherosclerosis, resulting in more diffuse coronary artery lesions and worse clinical outcomes [19, 20]. Nguyen et al. [21] found that endothelial dysfunction is also independently associated with asymptomatic myocardial ischemia in patients with T2DM. However, it remains to be elucidated whether the relationship between impaired endothelial function and the severity of coronary artery disease is influenced by the status of glycemic control in type 2 diabetic patients. In this study, we investigated the association of brachial artery FMD with angiographic significant coronary artery stenosis and atherosclerosis score in a cohort of type 2 diabetic patients according to the status of glycemic control assessed by blood concentration of glycated hemoglobin (HbA1c).

## Methods

This study complied with the Declaration of Helsinki. The study protocol was approved by the local hospital ethics committee, and written informed consent was obtained from all participants.

### Study population

**Fig. 1:**
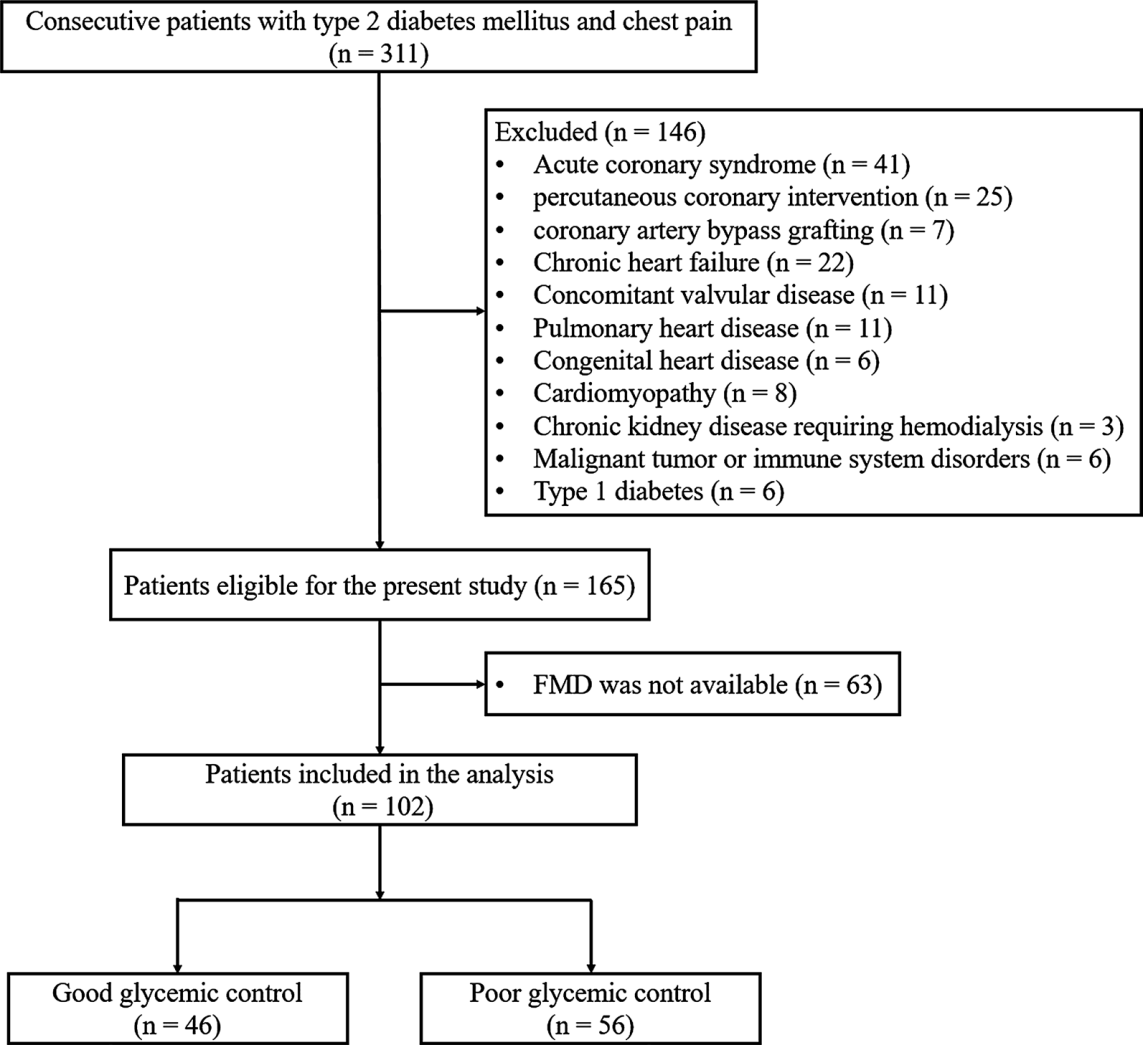
Flowchart of patient enrollment. FMD, flow-mediated dilation

A total of 311 consecutive patients with T2DM and chest pain on exertion referred for diagnostic coronary angiography from January 2018 to May 2019 were enrolled. The diagnosis of T2DM was made according to the criteria of American Diabetes Association [22]. Hypertension and dyslipidemia were diagnosed according to seventh report of the Joint National Committee on prevention, detection, evaluation, and treatment of high blood pressure (JNC 7) and guideline of the National Cholesterol Education Program (ATP III), respectively [23, 24]. For the purpose of research, patients with acute coronary syndrome (n = 41), a history of coronary revascularization (coronary artery bypass grafting: n = 7; percutaneous coronary intervention: n = 25), chronic heart failure (n = 22), concomitant valvular disease (n = 11), pulmonary heart disease (n = 11), congenital heart disease (n = 6), or cardiomyopathy (n = 8) were excluded. We also excluded patients who had chronic kidney disease requiring hemodialysis (n = 3) and those who had malignant tumor or immune system disorders (n = 6). Patients with type 1 diabetes were excluded by measurement of C-peptide (n = 6) [22]. Sixty-three patients were further excluded due to unavailability of FMD. Thus, the remaining 102 patients were enrolled in the final analyses. To evaluate the influence of glycemic control on FMD and coronary artery disease, T2DM patients were arbitrarily categorized as good glycemic control (n = 46, HbA1c < 7.0%) and poor glycemic control (n = 56, HbA1c ≥ 7.0%). Baseline demographics, risk factors for coronary artery disease, and medications of all patients were recorded (Fig. 1).

### Coronary angiography

Coronary angiography was performed through radial or femoral approach. Quantitative coronary angiography was performed using the Cardiovascular Measurement System version 3.0 software (Terra, GE, USA) by two interventional cardiologists who were blinded to the study protocol. Significant coronary artery disease was diagnosed if luminal diameter narrowing was estimated as ≥ 50% in a major epicardial coronary artery. Left main coronary artery stenosis ≥ 50% was considered as 2-vessel disease. The SYNTAX score and Gensini score were calculated and used as indexes of the anatomic extension and severity of coronary atherosclerosis [25, 26].

### Brachial artery FMD measurement

Brachial artery FMD was assessed in the right arm using a high-resolution ultrasound machine with a 10‐MHz linear array probe and the GE Vivid 7 Imaging System following the recommended protocol [3]. Briefly, a blood pressure cuff was inflated on the forearm for 5 min at 200 mmHg and the change in brachial artery diameter was measured and recorded for 3 min following cuff deflation. Brachial artery diameters were determined during end-diastole (gated with electrocardiogram R wave) by measuring the distance between the near and far wall of the intima. FMD expressed as a maximal percentage increase in brachial artery diameter from baseline was recorded. All brachial artery FMD procedures were performed by single experienced ultrasound technician, and images were measured blind to patient data and study design.

### Statistical analysis

Continuous variables are presented as mean ± standard deviation (SD) and median (25th–75th percentile) for normal and non-normal distribution, respectively, and categorical data are summarized as frequencies (percentages). We evaluated the differences in categorical variables between groups with a chi-square test. We evaluated the normality of distribution with the Kolmogorov–Smirnov test and applied logarithmic or square-root transformations to continuous variables showing a non-normal distribution. The differences between groups for continuous variables were analyzed by Student t test. We constructed multivariable logistic regression models to assess the independent determinants of coronary artery disease without (Model 1) and with (Model 2) FMD. All analyses used 2-sided tests with an overall significance level (alpha) of 0.05, and all tests were performed with SPSS 25.0 for Windows (SPSS, Inc., Chicago, IL, USA).

## Results

### Clinical characteristics of the population

Patients with poor glycemic control were older (P = 0.026) and exhibited higher serum levels of fasting blood glucose (P = 0.009), HbA1c (P < 0.001) and high-sensitivity C- reactive protein (hsCRP) (P = 0.002) compared to those with good glycemic control. However, there were no significant differences between the two groups in gender distribution, body mass index, proportion of cigarette smoking and hypertension, brachial blood pressure, kidney function and lipid profiles (all P > 0.05) (Table 1).

**Table 1:** Baseline characteristics of diabetic patients with good and poor glycemic control

	Good glycemic control group (n = 46)	Poor glycemic control group (n = 56)	*P value*
Male, n (%)	33 (71.7)	40 (71.4)	0.972
Age, years	61.83 ± 9.34	65.43 ± 6.73	0.026
Body mass index, Kg/m^2^	28.41 ± 2.59	28.16 ± 2.55	0.622
Cigarette smoking, n (%)	17 (37.0)	22 (56.4)	0.810
Hypertension, n (%)	30 (65.2)	45 (80.4)	0.085
Systolic blood pressure, mm Hg	135.35 ± 19.01	141.77 ± 18.69	0.090
Diastolic blood pressure, mm Hg	74.85 ± 10.69	77.82 ± 12.42	0.204
Fasting blood glucose, mmol/L	6.03 ± 0.81	6.90 ± 2.08	0.009
HbA1c, %	6.53 ± 0.31	8.13 ± 1.29	< 0.001
Serum creatinine, μmol/L	77.04 ± 15.21	78.70 ± 17.55	0.615
Serum uric acid, μmol/L	345.76 ± 79.73	345.98 ± 82.74	0.989
GFR, mL/min/1.73m^2^	86.83 ± 14.70	83.29 ± 14.47	0.225
Triglyceride, mmol/L	1.39 ± 0.84	1.92 ± 1.75	0.064
Total cholesterol, mmol/L	3.84 ± 0.88	3.89 ± 1.06	0.810
HDL cholesterol, mmol/L	1.23 ± 0.28	1.14 ± 0.25	0.100
LDL cholesterol, mmol/L	2.25 ± 0.80	2.21 ± 0.74	0.804
Apolipoprotein A, g/L	1.24 ± 0.16	1.17 ± 0.19	0.076
Apolipoprotein B, g/L	0.75 ± 0.33	0.75 ± 0.20	0.975
Lipoprotein (a), g/L	0.25 ± 0.27	0.33 ± 0.35	0.220
hsCRP, mg/L	1.18 (0.47–3.04)	2.16 (1.19 ~ 5.76)	0.002
Presence of CAD, n (%)	17 (37.0)	38 (67.9)	0.002
SYNTAX score	7.28 ± 10.54	15.88 ± 16.36	0.003
Gensini score	15.07 ± 21.03	48.99 ± 48.88	< 0.001
Medication, n (%)			
ACE inhibitors/ARBs	25 (54.3)	27 (48.2)	0.538
β-blockers	28 (60.9)	40 (71.4)	0.260
Calcium channel blockers	13 (28.3)	14 (25.0)	0.710
Statins	43 (93.5)	49 (87.5)	0.312
Flow mediated dilation, %	6.94 ± 2.20	6.03 ± 2.08	0.036

Values are given as mean ± standard deviation (SD), median (25th–75th percentile) or number (percentage)

*ACE* angiotensin converting enzyme, *ARB* angiotensin receptor blocker, *BUN* blood urea nitrogen, *CAD* coronary artery disease, *GFR* glomerular filtration rate, *HbA1c* glycated hemoglobin, *HDL* high-density lipoprotein, *hsCRP* high-sensitivity C-reactive protein, *LDL* low-density lipoprotein

### Angiographic and FMD findings

**Fig. 2:**
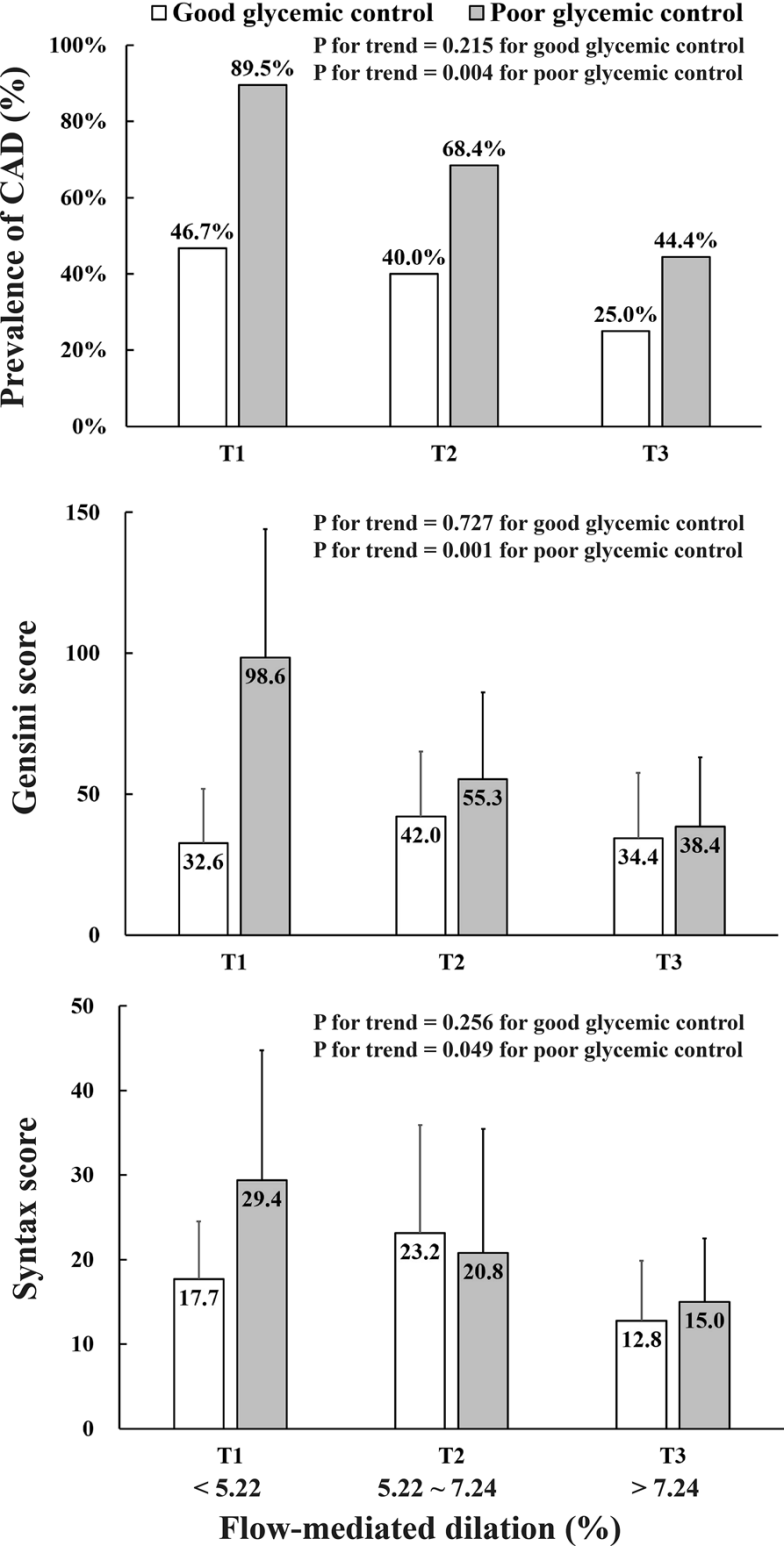
Significant decrease in the prevalence and severity of coronary artery disease (CAD) across tertiles of brachial artery flow-mediated dilation (FMD) in type 2 diabetic patients with good or poor glycemic control

Overall, the prevalence of significant coronary artery disease was higher (67.9% vs. 37.0%, P = 0.002) and the severity of coronary atherosclerosis was more prominent (Gensini score: 48.99 ± 48.88 vs. 15.07 ± 21.03, P < 0.001; SYNTAX score:15.88 ± 16.36 vs. 7.28 ± 10.54, P = 0.003) in patients with poor glycemic control than those with good glycemic control. Consistently, FMD was also significantly lower in patients with poor than those with good glycemic control (6.03 ± 2.08% vs. 6.94 ± 2.20%, P = 0.036). We arbitrarily categorized FMD values of all patients into three groups according to tertile of FMD (< 5.22%, 5.22–7.24% and > 7.24%). In patients with poor glycemic control (HbA1c ≥ 7.0%), a significant decrease in the prevalence of significant coronary artery disease, Gensini score and SYNTAX score was observed across the three tertiles of FMD (all P for trend < 0.05), while no such trends were found in those with good glycemic control (all P for trend > 0.05) (Fig. 2).

### Multivariate regression analysis

Multivariate regression analyses were performed to analyze the association between coronary artery disease and FMD in two glycemic control groups. After adjustment for confounding risk factors (age, gender, hypertension, smoke, total-to-high-density lipoprotein cholesterol ratio, GFR and hsCRP), tertile of FMD (OR = 0.227 95% CI 0.056–0.915, P = 0.037) remained to be independent determinants for coronary artery disease in patients with poor glycemic control (Model 2B) (Table 2). Compared with model 1B, the addition of tertile of FMD in model 2B significantly improved the goodness-of-fit and predictive performance with an increase of Nagelkerke R^2^ of 8.0% (P = 0.018). In multiple linear regression analysis, tertile of FMD was independently correlated with Gensini score (β = − 0.470, P < 0.001) and SYNTAX score (β = − 0.349, P = 0.004), respectively, in patients with poor glycemic control (Table 3). However, these result patterns were not found in patients with good glycemic control (Tables 2 and 3).

**Table 2:** Multivariate logistic regression analyses for coronary artery disease in diabetic patients with good and poor glycemic control

Variables		Good glycemic control group			Poor glycemic control group	
		OR (95% CI)	P value		OR (95% CI)	P value
	Model 1A			Model 1B		
Male		1.492 (0.137–16.197)	0.742		8.47 (0.6–119.56)	0.114
Age		0.973 (0.874–1.084)	0.622		0.953 (0.806–1.126)	0.573
Body mass index		0.882 (0.585–1.328)	0.547		1.592 (0.954–2.656)	0.075
Smoke		1.800 (0.372–8.719)	0.465		3.588 (0.573–22.482)	0.172
Hypertension		7.320 (1.167–45.933)	0.034		3.332 (0.435–25.548)	0.247
Total-to-HDL cholesterol ratio		0.983 (0.503–1.923)	0.960		8.945 (2.004–39.919)	0.004
GFR		0.962 (0.898–1.030)	0.269		0.948 (0.866–1.037)	0.243
Log-transferred hsCRP		0.984 (0.539–1.795)	0.958		1.415 (0.701–2.857)	0.333
	Model 2A			Model 2B		
Male		2.668 (0.187–38.168)	0.470		14.289 (0.707–288.728)	0.083
Age		0.949 (0.849–1.061)	0.358		0.934 (0.774–1.126)	0.473
Body mass index		0.970 (0.622–1.513)	0.894		1.753 (0.943–3.258)	0.076
Smoke		2.408 (0.458–12.679)	0.300		3.725 (0.501–27.674)	0.199
Hypertension		8.476 (1.202–59.789)	0.032		5.577 (0.470–66.239)	0.173
Total-to-HDL cholesterol ratio		1.158 (0.562–2.389)	0.691		11.882 (1.713–82.402)	0.012
GFR		0.964 (0.899–1.034)	0.303		0.941 (0.847–1.046)	0.261
Log-transferred hsCRP		0.815 (0.423–1.570)	0.540		1.015 (0.462–2.228)	0.971
Tertiles of FMD		0.410 (0.128–1.307)	0.132		0.227 (0.056–0.915)	0.037

Values are odds ratios (95% confidence interval)

*FMD* flow-mediated dilation, *GFR* glomerular filtration rate, *HDL* high-density lipoprotein, *hsCRP* high-sensitivity C-reactive protein

**Table 3:** Linear regression models of Gensini score and Syntax score for diabetic patients with good and poor glycemic control

Variables	Gensini score				SYNTAX score			
	Good glycemic control		Poor glycemic control		Good glycemic control		Poor glycemic control	
	Beta	P value	Beta	P value	Beta	P value	Beta	P value
Male	0.044	0.849	0.170	0.159	0.024	0.918	0.204	0.105
Age	− 0.174	0.398	0.217	0.074	− 0.181	0.374	0.227	0.073
Body mass index	0.022	0.926	0.076	0.522	− 0.017	0.940	0.096	0.436
Smoke	0.088	0.591	0.237	0.039	0.124	0.445	0.270	0.024
Hypertension	0.440	0.006	0.093	0.369	0.431	0.007	0.069	0.521
Total-to-HDL cholesterol ratio	0.114	0.458	0.326	0.004	− 0.047	0.756	0.394	0.001
GFR	− 0.227	0.264	0.101	0.405	− 0.262	0.193	− 0.052	0.678
Log-transferred hsCRP	0.297	0.100	0.185	0.100	0.280	0.115	0.081	0.481
Tertiles of FMD	− 0.088	0.595	− 0.470	< 0.001	− 0.126	0.444	− 0.349	0.004

*FMD* flow-mediated dilation, *GFR* glomerular filtration rate, *HDL* high-density lipoprotein, *hsCRP* high-sensitivity C-reactive protein

## Discussion

The results of the present study showed that type 2 diabetic patients with poor glycemic control had lower FMD and more severe coronary artery disease than those with good glycemic control. Decreased FMD was an independent determinant of coronary artery disease and this association was more prominent in type 2 diabetic patients with poor glycemic control. These findings emphasized the importance of glycemic control in patients with T2DM.

Endothelial dysfunction is an initial manifestation in the development of atherosclerosis and also participates in plaque progression [12, 27]. It is mainly characterized by a reduction in the bioavailability of nitric oxide (NO) and excess production of reactive oxygen species [5, 6]. Many cardiovascular risk factors contribute to endothelial dysfunction, and major elements in diabetes (such as hyperglycemia, insulin resistance, and dyslipidemia) play an important role in the pathogenesis of endothelial dysfunction, resulting in an aggravated pro-atherogenic phenotype [12, 13]. Moreover, the diabetic milieu yields an impairment of metabolic environment homeostasis represented by chronic inflammation, oxidative stress, pro-coagulability, impaired fibrinolysis, and neovascularization abnormalities that cumulatively alter vascular structure [16, 27–29]. These pathophysiologic features, together with increased expression of pro-inflammatory cytokines, lead to abnormal endothelium-dependent vasodilation which could be investigated using FMD of the brachial artery.

The major finding of our study is that declined FMD is associated with coronary artery disease, and this association is more prominent in T2DM patients with poor glycemic control. Lower FMD is also an independent determinant of coronary artery disease in diabetic patients with poor glycemic control. These results support the notion that poor glycemic control is closely related to severe endothelial dysfunction, which aggravates the process and outcome of atherosclerosis [30]. Our results are consistent with previous studies regarding the relation of T2DM with endothelial dysfunction detected by FMD [12], and further substantiate the importance of pro-inflammatory cytokines and metabolic disturbance in the development of diabetic micro- and macro-vascular complications [31, 32], detrimental left ventricular remodeling as well as adverse cardiovascular outcome [33, 34]. These findings also highlight that endothelial dysfunction may be a critical early target for preventing atherosclerosis in patients with T2DM as abnormalities in vascular reactivity and biochemical markers of endothelial cell activation are present early in individuals at risk of developing T2DM, and repair of plaque rupture and reendothelialization of coronary lesions are often asymptomatic or silent in most cases [35].

In this perspective, our observations have important clinical relevance. Previous studies have shown that treatment with angiotensin-converting enzyme inhibitors or angiotensin-receptor blockers is associated with improvement of endothelial function and reduced incidence of new-onset diabetes possible through increased serum bradykinin levels which improves insulin-mediated glucose uptake and endothelial function, increased vascular NO activity, and reduced vascular inflammation [12, 36]. Lipid-lowering agents particularly statins not only decrease serum low-density lipoprotein (LDL) cholesterol and hsCRP levels and risk of cardiovascular disease, but also substantially improve the clinical outcome of patients with coronary artery disease. In addition, several studies have demonstrated an improvement in endothelial function before the reduction in serum cholesterol levels [37, 38]. However, it should be noted that statin use may increase fasting blood glucose and HbA1c levels due to increased insulin resistance or reduce insulin release, which could contribute to the genesis of diabetes [35]. Proprotein convertase subtilisin/kexin type 9 (PCSK9) inhibitors have been shown to dramatically reduce plasma LDL cholesterol and cardiovascular risk in atherosclerotic disease patients, without increasing the risk of new-onset diabetes and worsening glycemia [39].

Despite conflicting results in the relationship of HgbA1c to cardiac outcomes and survival, efforts have been made to achieve optimal glycemic control for patients with T2DM because hyperglycemia is a major causal factor in the development of endothelial dysfunction and chronic diabetic vascular complications through multiple mechanisms [12, 40]. Sara and co-workers reported that microvascular dysfunction is associated with poor glycemic control amongst female diabetics with non-obstructive coronary artery disease, which results in reduced myocardial flow reserve and microvascular angina [41]. Oral hypoglycemic agents have been shown to play a role in improving endothelial function beyond their glycemic control [40, 42, 43]. FMD was improved after 3 months of treatment with metformin [42]. In a single-arm study, sitagliptin, a dipeptidyl peptidase-4 inhibitor, showed an improvement in FMD measurements in moderately controlled patients with T2DM, in addition to a decrease in HbA1c within 2 weeks of therapy [44]. Sodium-glucose cotransporter 2 (SGLT2) inhibitors are a relatively new class of oral anti-diabetic agents with promising cardiovascular benefits, including a slight reduction in blood pressure and arterial stiffness [45–47]. The DEFENSE study demonstrated that with dapagliflozin add-on therapy to metformin for 16 weeks, vascular endothelial function as assessed by FMD was significantly ameliorated in patients with inadequately controlled early stage T2DM, and such a beneficial effect was due to improved oxidative stress [48]. In the EDIFIED trial, a 12-week therapy with dapagliflozin, in addition to insulin and metformin therapies, resulted in significant reductions in HbA1c, fasting blood glucose, and surrogate markers of endothelial function. Furthermore, there was a significant association between reduction in HbA1c and improvement in FMD in the dapagliflozin group [49]. These observations together with our present findings should have an impetus on further investigations regarding the protective role of new anti-diabetic agents especially SGLT2 on endothelial function and their ability to suppress the progression of coronary atherosclerosis.

## Limitation

We recognize that there are certain limitations in this study. First, the study is cross-sectional for the point of FMD and coronary artery disease investigation, thereby allowing us to detect association, not to establish a causative link of FMD with coronary atherosclerosis and to predict clinical outcome. Nevertheless, prescription data were accurately captured by using standard database software, thus these results reflect true associations in the real-world setting. Second, only single measurement of baseline HbA1c level was used, which may not accurately reflect the exact status of glycemic control during follow-up. Recent studies have shown an advantage of measuring visit-to-visit HbA1c variability (i.e., long-term glycemic variability) in the assessment of risk of cardiovascular disease for patients with T2DM [50–52]. Likewise, the duration of T2DM was not determined, but impairment of endothelium-dependent vasodilation is independently associated with long-term diabetic duration [53]. Finally, it is possible that the prevalence and course of coronary artery disease could also be influenced by the medical treatments and other risk factors.

## Conclusion

This study indicates that poor glycemic control exerts an adverse effect on endothelial function, and aggravates coronary atherosclerosis in T2DM. These observations, at least partly, provide substantial insight into the management of type 2 diabetic patients with coronary artery disease.

## Data Availability

The datasets used and/or analyzed during the current study are available from the corresponding author on reasonable request.

## Abbreviations

FMD: Flow-mediated dilation
GFR: Glomerular filtration rate
HbA1c: Glycated hemoglobin A1c
HDL: High-density lipoprotein
SGLT2: Sodium-glucose cotransporter 2
T2DM: Type 2 diabetes mellitus
